# Non-Isothermal Simulation and Safety Analysis of Twin-Screw Extrusion Process for Synthetizing Glycidyl Azide Polymer-Based Energetic Thermoplastic Elastomer

**DOI:** 10.3390/polym15183662

**Published:** 2023-09-05

**Authors:** Junming Yuan, Yan Liu, Jinying Wang, Yuan Qu, Hu Sun, Yue Qin, Nan Wang

**Affiliations:** 1 School of Environment and Safety Engineering, North University of China, Taiyuan 030051, China; 18635820488@163.com (Y.L.); wjywzhy@126.com (J.W.); sunhudyx@163.com (H.S.); cysl080579@163.com (Y.Q.); xiangbei0621@163.com (N.W.); 2 Jinxi Industrial Group Co., Ltd., Taiyuan 030027, China; 13593198497@163.com

**Keywords:** GAP-ETPE, mechanical sensitivity, conveying element, safety, non-isothermal, simulation

## Abstract

In order to study the temperature variation and flow characteristics in the twin-screw reactive extrusion process of synthetizing glycidyl azide polymer-based energetic thermoplastic elastomer (GAP-ETPE), a non-isothermal simulation and a safety analysis were carried out. Firstly, based on the synthesis principle of GAP-ETPE, a mechanical sensitivity test, viscosity test and differential scanning calorimetry (DSC) of GAP-ETPE were carried out. Secondly, a three-dimensional physical model of the intermeshing co-rotating conveying element was established by Gambit. A three-dimensional non-isothermal numerical simulation of the conveying and kneading elements was carried out using FLUENT 19.0 software. The temperature, pressure and shear stress field of conveying and kneading elements with different staggered angles were analyzed and compared. The results show that the maximum temperature of the kneading element is always slightly higher than that of the conveying element at the same rotational speed, but the average temperature in the flow channel is always slightly higher than that of the kneading element. The inlet and outlet pressure difference of the kneading elements with a 90° offset angle is the smallest and the safety is the highest. The shear stress in the flow channel of the conveying element is higher than that of the kneading element as a whole, but the shear stress near the outlet of the 90° kneading element is higher than that in the flow channel of the conveying element. Among the kneading elements, the 90° kneading element has the strongest dispersing and mixing ability, followed by the 60° and 45° kneading elements. According to the thermal and physical parameters of the material, the ignition response time is approximately 6 s, which provides a theoretical guide for the safety design of the GAP-ETPE twin-screw extruder.

## 1. Introduction

Energetic thermoplastic elastomer (ETPE) has the advantages of excellent mechanical properties, high energy, low sensitivity and repeatability, making it an important material base for the development of high-performance explosives. Currently, ETPE has been used in the polyolefin 13 blended explosive and has been applied to the high-energy propellant, increasing its low-temperature impact strength by more than 120% [1]. The application of a high solid content modified the double-base solid propellants and has significantly improved their low-temperature mechanical properties, and ETPE also has good application prospects in combustible cartridges. However, the current synthesis of ETPE uses the intermittent reaction kettle method, which has problems, such as an unstable product quality, poor interbatch repeatability, low production efficiency and low production capacity, making it difficult to meet the urgent demand for ETPE for new high-performance explosives. Therefore, research should be conducted on the reactive extrusion synthesis process of energetic thermoplastic elastomers to improve the quality of ETPE, shorten its production cycle and rapidly promote the development and application of high-performance explosives, which is also an urgent need for the development of weapons and equipment. The development of solid propellant formulations using various energetic and non-energetic binders with a little emphasis on ETPEs was reviewed by M. L. Chan et al. [2]. The review paper explains the synthesis of a wide range of ETPEs, their propellant and explosive formulations along with the thermal studies of the same. A. G. Stern and H. G. Adolph patented the synthesis process of hydrolysable ETPEs based on BAMO, AMMO and poly caprolactone-poly formal polymers [3]. The advantage of this type of ETPE is to recover the ingredients of propellant, explosive or pyrotechnic compositions at the end of their life cycle by hydrolyzing the binder instead of melting.

Glycidyl azide polymer-based energetic thermoplastic elastomer (GAP-ETPE) is a kind of thermoplastic elastomer, which is synthesized by the end capping reaction of GAP with diisocyanate followed by a chain extension with diol. As one of the most-studied compounds in high-energy prepolymers, GAP not only significantly improves the burn rate of propellants but GAP-ETPE-based propellants also have better creep resistance than conventional thermoset propellants [4]. So, GAP-ETPE has attracted much attention from domestic and foreign energy materials workers [5]. The intermittent co-rotating screw extruder is one of the most commonly used types of polymer compounding equipment, and the variation in the screw extruder process parameters plays a crucial role in the quality of the GAP-ETPE products and the safety of the equipment. In addition, the design of an advanced control system for the intermittent reaction process has enabled the successful application of a non-self-compensating dynamic matrix control method to the reactor experimental setup [6].

Reactive extrusion (REX) is a manufacturing technique that combines traditional melt extrusion with chemical reactions, including polymerization, polymer functionalization and depolymerization (chemical recycling) [7,8].The transfer screw extruder (TSE) is a key piece of equipment in polymer processing, mainly used as a conveyor, mixer or reactor for particles, granules and viscous fluids [9]. The unique geometry of screw extruders allows the handling of very viscous media; thus, they can be used as continuous reactors to conduct polymerization reactions without using any solvents. This is called reactive extrusion polymerization [10]. Sobhani, H. and Verma, S. K. et al. [11,12] combined the virtual domain method with the finite element method to study the flow characteristics of polymer melt in the conveying zone of an intermeshing rotating twin-screw extruder under isothermal conditions. With the development of computational methods, more and more researchers are using numerical simulation to study the non-isothermal flow mechanism in TSEs [13,14,15,16]. Ishikawa T. et al. [17] conducted a three-dimensional non-isothermal simulation study of the kneading zone of the co-rotating twin-screw extruder at different speeds and compared the simulation results with the experimental observation to verify the accuracy of the simulation. In addition, Khalifeh et al. [18] investigated the numerical application of non-isothermal flows in a two-channel single-screw extruder using the finite volume method. Therefore, in order to achieve the expected quality throughout the manufacturing process of energetic materials, it is necessary not only to analyze the raw materials and process parameters [19,20] but also to pay special attention to the mechanical properties of these compounds during the extrusion process [21]. The current polymer materials and processing industry heavily rely on single-screw and twin-screw extrusion technology. In order to promote the upgrading of circular economy technology, bridging experimental characterization technology and the predictive ability of modeling and software tools are essential [22].

In order to investigate the effects of operating parameters and screw geometry on the mixing performance during the reactive extrusion process in the TSE, many experimental and numerical simulation studies have been carried out in recent years [23,24,25]. Ozkan, S. et al. [26] developed a 7.5 mm twin-screw extruder to process high-energy formulations containing nanoparticles. The twin-screw extrusion process has been shown to have several inherent advantages. Rozen [27] used the product distribution of acid-base neutralization and ester hydrolysis to characterize the mixing process of a co-rotating twin-screw extruder. Berzin, F. et al. [28] chose transesterification to optimize and scale up the twin-screw reactive extrusion process. Ke, Z. et al. [29] used the finite element method to analyze the distribution and variation in viscosity during the single-screw extrusion of propellants and studied the threshold and distribution of sensitive parameters, such as pressure and temperature. Zhu, L. and Cegla, M. et al. [30,31] analyzed the polymerization of E-caprolactone in a co-rotating twin-screw extruder using three-dimensional numerical simulation. It is concluded that viscous heat plays an important role in energy generation when the system has high viscosity. Zhang et al. [32] prepared a polypropylene (PP)/wood-fiber (WF) composite foam using the pressure quenching batch method and investigated the effects of the screw configuration, screw speed and silica on the physical and mechanical properties and foaming properties of PP/WF composites. Zong et al. [33] studied the flow pattern, molecular weight distribution and temperature of poly (p-phenylenediamine terephthalate) in three extruders with different elements. Zhang et al. [34] calculated the area draw ratio, instantaneous efficiency and time average efficiency of the mixing disc by studying the distributed mixing power and simulated the local residence time distribution of the TSE using the particle tracking method. Rathod et al. [35] carried out a three-dimensional finite element simulation of a carboxy methyl cellulose aqueous solution in the mixing zone of a twin-screw mixer and studied the influence of the speed, flow rate and blade angle of the mixer on the dispersive mixing of the twin-screw mixer in order to better understand the relationship between the flow, mixing and reaction during polymer preparation. Sayin, Hirata, Hetu and Sun et al. [36,37,38,39] carried out three-dimensional numerical simulations in a TSE to investigate the effects of the screw speed, mixing block dislocation, inlet flow rate and stress on the mixing and reaction processes in a TSE. Jong-Sung You et al. [40] characterized the GAP/polyol ETPEs using DSC, dynamic mechanical analysis (DMA) and rheological mechanics. The results show that with an increasing hard segment content both GAP/PTMG ETPEs and GAP/PCL ETPEs exhibit microphase separation transitions.

In the process of mixing energetic materials, the temperature is a key factor affecting the safety of the mixing. If the speed of the impeller is low, the viscosity of the material will be relatively high, the mixing will be inadequate and the expected effect will not be achieved. If the rotating speed of the TSE is high, the viscous heat and shear friction of the material will be greater, the temperature in the channel will be higher, the possibility of combustion and explosion accidents will be greater and the safety will be worse. At the same time, excessive temperatures can volatilize materials and produce flammable gases, which can lead to combustion and/or explosion accidents. In the mixing process, the temperature of the material must be strictly controlled to avoid large temperature fluctuations. Therefore, it is necessary to simulate the temperature field of the flow channel and use the local maximum temperature in the flow channel to characterize the safety of mixing. The main purpose of this paper is to assess the safety of the gauge pressure and critical load pressure obtained from friction susceptibility tests by comparing them with the squeezing pressure and shear stress to which the fluid is subjected in the simulation.

Based on the rheology of the polymer flow, the temperature field, pressure field and shear stress field of the non-Newtonian melt of GAP-ETPE flowing in the twin-screw reactor were analyzed in order to understand the effect of geometric and operational parameters on the reactive extrusion process. In this paper, the GAP-ETPE synthesis process, its performance characterization and a mechanical sensitivity test are carried out. The thermal degradation characteristics of GAP-ETPE are analyzed and the mechanical sensitivity results are obtained. Meanwhile, the three-dimensional physical model of the intermeshing co-rotating conveying element is established using Gambit 2.4 software. Under non-isothermal simulation conditions, the temperature field, pressure field and shear stress field are simulated and analyzed by FLUENT 19.0 software, taking into account factors such as friction, viscous heat generation and synthetic reaction heat. The material flow and temperature changes of GAP-ETPE in the process of conveying and kneading element reactive extrusion are studied using the three-dimensional numerical simulation method; the differences and relationships of the pressure, temperature and shear stress are analyzed; the effects of the screw speed and kneading element pitch angle on the reactive extrusion are discussed; and the ignition response time is calculated according to the thermo-physical parameters of the materials and the convergence of the calculation results. This study provides theoretical guidance for the safety design of the GAP-ETPE twin-screw extruder.

## 2. Materials and Methods

### 2.1. Synthesis and Characterization of GAP-ETPE

#### 2.1.1. Synthesis Reaction of GAP-ETPE

**Scheme 1:**
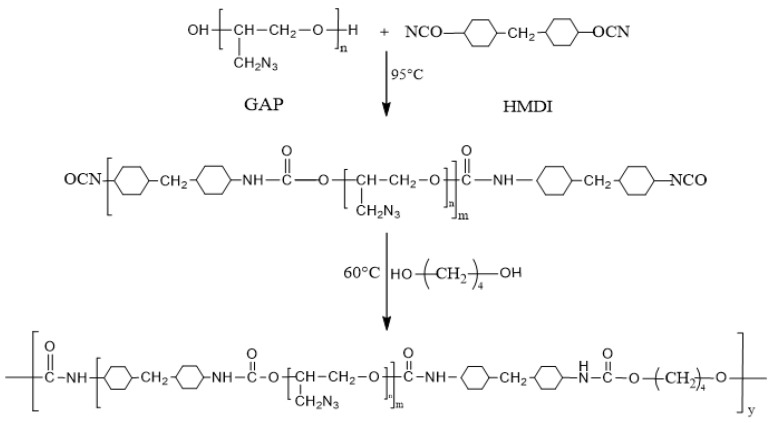
Synthesis process of GAP ETPE.

**Figure 1:**
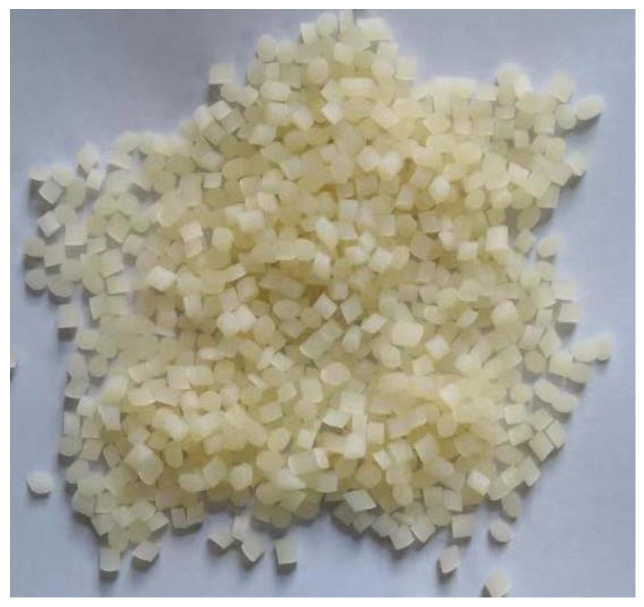
Diagram of a sample of granular GAP-ETPE synthesis product.

GAP-ETPE, Xi’an 204 Institute, Industrial Grade, Shaanxi, China. The synthesis process is shown in Scheme 1. The isocyanate is stored in tank A and the dehydrated polyols, chain extenders and other additives are fully mixed and stored in tank B. The materials are accurately measured and mixed in proportion by the casting machine and then fed into the feed port of the twin-screw reaction extruder. After the twin-screw reaction extruder, the strip-shaped samples are extruded and cut into granules via water-cooled drawing. After forming, the grain is post-cured at room temperature or a high temperature (80 °C or less). The prepared specimen is shown in Figure 1.

#### 2.1.2. Performance Characterization and Sensitivity Testing of GAP-ETPE

HTC-1 DSC: Beijing Hengjiu Experimental Equipment Co., Ltd., Beijing, China. The start temperature is set at 30 °C, the end temperature is set at 380 °C, the heating rate is 5 °C/min, the DTA resolution is 0.01 μV, and the sample size is 5–6 mg, with a measurement accuracy of ±2%.

Viscosity test: R/S CPS rheometer, Shanghai Beide Digital Technology Co., Ltd., Shanghai, China. The explosive was loaded into a sealed bottle and dispersed in an ultrasonic constant temperature oil bath at 120 °C for 2 h to form a suspension. The rheological properties of the suspension were then tested using the constant rotation measuring block in the R/S CPS rheometer. The temperature was 120 °C, the shear rate was 2 s^−1^, the number of test points was 60, and the test time was 60 s, rotational viscometer accuracy of ±0.1% and repeatability of ±0.2%.

Mechanical sensitivity: The shock sensitivity is tested according to GJB772A-1997 [41] with a dose of 30 mg, a drop height of 25 cm and a drop weight of 10 kg. The friction sensitivity is tested in accordance with GJB772A-1997 with a dose of 20 mg, a gauge pressure of 2.45 MPa and a swing angle of 66°.

### 2.2. Establishment of Finite Element Model

#### 2.2.1. Finite Element Model

**Figure 2:**
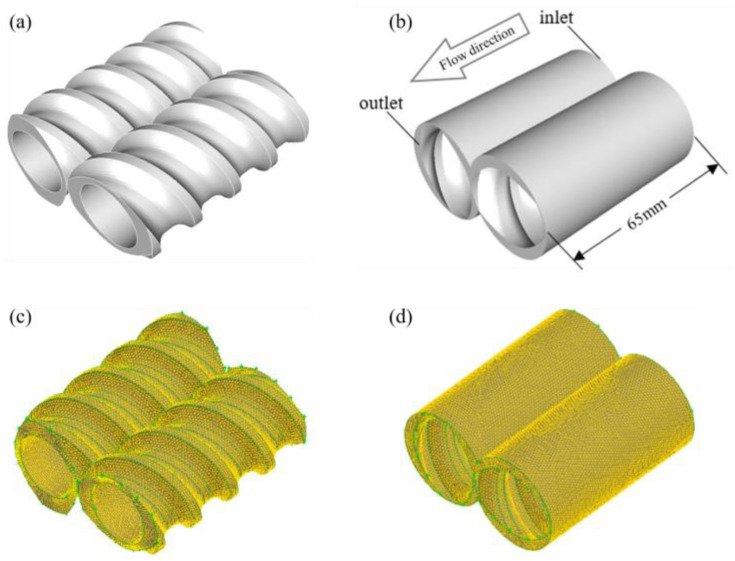
Screw geometry and finite element model: (**a**) screw geometry; (**b**) flow channel geometry; (**c**) finite element model of screw; (**d**) finite element model of flow channel.

The twin-screw extruder consists of conveying and kneading elements. Before using FLUENT for simulation calculations, the geometry model of the conveying and kneading elements must be created and meshed using Gambit 2.4 software. The modeling of the conveying element is based on the geometry of intermeshing co-rotating twin screws, and the flow channel model uses Boolean subtraction to remove the conveying element. The physical and finite element models of the screw geometry are shown in Figure 2.

**Figure 3:**
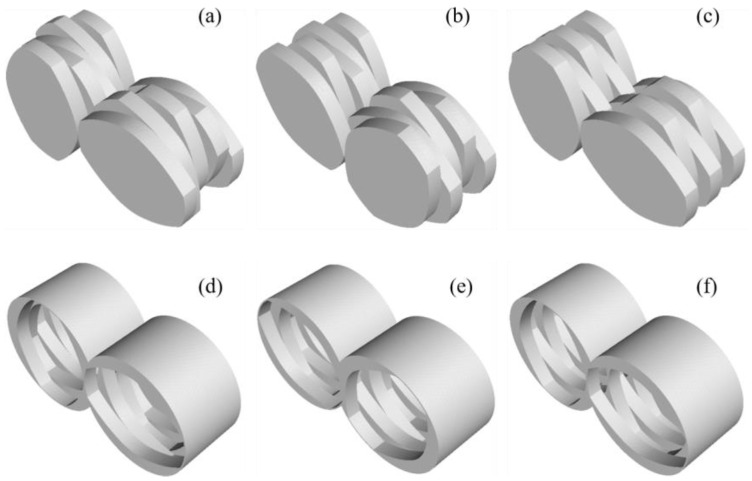
Geometry model of kneading element: (**a**) S45 geometry model; (**b**) S60 geometry model; (**c**) S90 geometry model; (**d**) geometric model of S45 flow channel (**e**) geometric model of S60 flow channel; (**f**) geometric model of S90 flow channel.

In order to compare the temperature changes of the conveying and kneading elements, the kneading elements with different pitch angles and the conveying elements were modeled separately. The screw parameters are given in Table 1. The kneading element is the main part of the mixing efficiency of the TSE and the main part of the temperature change in reactive extrusion. The pitch angle of the kneading element has a great influence on the temperature change. In this section, the typical stagger angles of 45°, 60° and 90° of kneading elements are selected to study effect of changes in the stagger angle on the extrusion temperature of the TSE reaction. Figure 3a,d are the kneading element and channel model with a stagger angle of 45°, i.e., S45; Figure 3b,e are the kneading element and channel model with stagger angle of 60°, i.e., S60; Figure 3c,f are the kneading element and channel model with stagger angle of 90°, i.e., S90. The kneading element parameters are given in Table 2.

#### 2.2.2. Mathematical Model

The extrusion movement of the fluid in the flow channel is very complex. Based on the flow characteristics of the polymer in the mixing section of the conveying element and the theory of fluid mechanics, the environment is assumed. The mathematical model of the flow channel is assumed to satisfy the following conditions: (1) the fluid is a power-law fluid; (2) the flow is laminar; (3) the fluid is an incompressible fluid; (4) the flow field is non-isothermal; (5) the viscous heat release of the material in the mixing process is considered; (6) the influence of inertia and gravity is neglected; (7) the non-slip condition is satisfied on the surface of the screw and the inner surface of the barrel.

**Figure 4:**
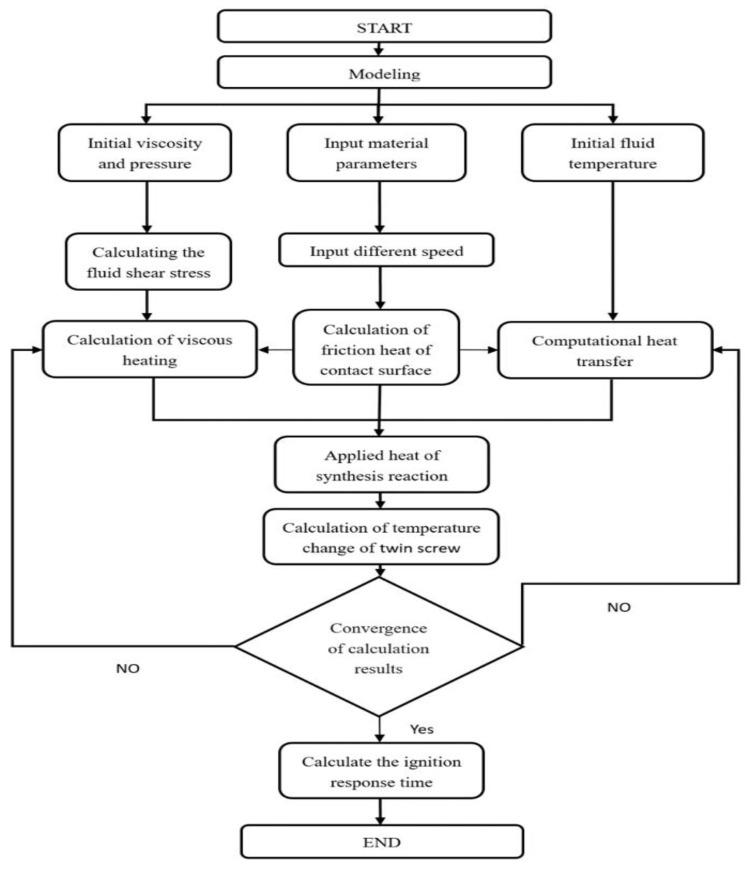
Flow chart of simulation calculation.

Based on the above assumptions, the flow chart of the numerical simulation is established as shown in Figure 4 below. However, the assumptions are relatively idealized, so the accuracy of the simulation results has a certain error compared to the experimental values.

The continuity equation is expressed as follows:

(1) $$\frac{u_{r}}{r}+\frac{\partial (u_{r})}{\partial r}+\frac{\partial (u_{\theta })}{\partial \theta }+\frac{\partial (u_{z})}{\partial z}=0$$

The momentum equations are expressed as follows:

(2) $$\tau_{xy}=2\mu \frac{\partial u_{x}}{\partial x}+\lambda \nabla \cdot \vec{u}$$

(3) $$\tau_{yy}=2\mu \frac{\partial u_{y}}{\partial y}+\lambda \nabla \cdot \vec{u}$$

(4) $$\tau_{zz}=2\mu \frac{\partial u_{z}}{\partial z}+\lambda \nabla \cdot \vec{u}$$

(5) $$\tau_{xy}=\tau_{yx}=\mu \left(\frac{\partial u_{x}}{\partial y}+\frac{\partial u_{y}}{\partial x}\right)$$

(6) $$\tau_{xz}=\tau_{zx}=\mu \left(\frac{\partial u_{x}}{\partial z}+\frac{\partial u_{z}}{\partial x}\right)$$

(7) $$\tau_{yz}=\tau_{zy}=\mu \left(\frac{\partial u_{y}}{\partial z}+\frac{\partial u_{z}}{\partial y}\right)$$

Where *μ* is the dynamic viscosity, Pa·s; *λ* is the second viscosity, Pa·s, usually −2/3; *p* is the pressure on the fluid microelement, Pa; and $\tau_{ij}$ is the stress tensor component, Pa.

Energy conservation refers to the rate of increase of the energy in the microelement body, which is equal to the net heat flux entering the microelement body plus the work performed on the microelement body by the mass force and the surface force. The expression is as follows:

(8) $$\rho c_{p}\left(\frac{\partial T}{\partial x}+\frac{\partial T}{\partial y}+\frac{\partial T}{\partial z}\right)=k\left(\frac{\partial^{2}T}{\partial x}+\frac{\partial^{2}T}{\partial y}+\frac{\partial^{2}T}{\partial z}\right)+\frac{1}{2}\eta \dot{\gamma }$$

Where *p* is the pressure, Pa; ρ is the density, g/${cm}^{3}$; *k* is the thermal conductivity, W/(m·K); $c_{p}$ is the specific heat capacity, J/(kg·K); *η* is the viscosity, Pa·s; and $\dot{\gamma }$ is the shear rate, s^−1^.

**Figure 5:**
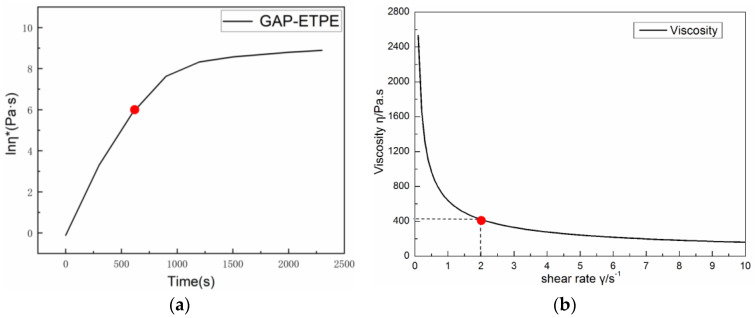
(**a**) Relationship between ${ln\;\eta }^{*}$ of GAP-ETPE versus time; (**b**) power-law fluid model.

In order to reduce the reaction time and speed up the reaction rate, the amount of catalyst added in the twin-screw reaction process is higher than the conventional amount. Since most of GAP-ETPE is in a viscous state throughout the twin-screw reaction process, the viscosity of GAP-ETPE is taken as the mean value. Figure 5a shows the relationship between the ${ln\;\eta }^{*}$ of GAP-ETPE and time, with a median of 6 selected for $ln\;\eta^{*}$, as shown by the red dot in Figure 5a. Therefore, in this simulation, the material property value of GAP-ETPE is selected as follows: $c_{p}=2223\;J/(kg\cdot K)$, $\rho =1183\;{kg/m}^{3}$, *η* = 400 Pa·s, as shown by the red dot in Figure 5b.

The viscosity of non-Newtonian fluids varies with the shear rate and shear stress, as shown in Figure 5b, so the ratio of shear stress to shear rate at a certain point on the flow curve is used to represent the viscosity at a certain value. This viscosity is called apparent viscosity, which is determined by *η* symbolic representation. The typical power-law model description commonly used for non-Newtonian fluids is shown in the following formula:

(9) $$\eta =k{\dot{\gamma }}^{n-1}$$

where *k* is the consistency index, also known as the consistency coefficient. The value of *k* is a measure of viscosity, but it is not equal to the viscosity value, and the higher the viscosity, the higher the value of *k*: *k* = 144.43 Pa·s. $\dot{\gamma }$ is the shear rate, s^−1^. The power-law exponent *n*, an index of the flow behavior or the non-Newtonian nature of the material, is a temperature-dependent parameter, and the greater the deviation of n from 1, the more non-Newtonian the material: *n* = 0.132. So, the final power-law fluid constitutive equation is *η* = 144.43${\dot{\gamma }}^{-0.868}$.

#### 2.2.3. Mesh Generation

Gambit 2.4 software was used to construct the three-dimensional model of the twin-screw extruder, in order to reduce the workload of simulation calculations. The inside of the twin screw was hollowed out, and the conveying element and the flow channel adopt a mixed mesh of tetrahedron and hexahedron. The interval size of the inner wall is 2, and the interval size of the thread contact surface of the conveying element and the flow channel is 1. To capture the flow characteristics in the gap, two layers of boundary layer are set on the outer wall of the channel, the gap is 0.4 mm and the growth factor is 1.2. The finite element model of the flow channel consists of 199,806 elements and 47,433 nodes. The finite element model of a screw consists of 276,775 elements and 65,706 nodes. The kneading elements with different staggered angles use the same mesh generation method and mesh density.

#### 2.2.4. Boundary Conditions

The rotation of the impeller causes the instability to grow in the fluid. The time selection is transient, and the dynamic mesh is set for the region of the impeller. To account for the heat generated by wall friction during rotation, a roughness constant of 0.5 and a roughness height of 10 mm are set on the contact surface, and the initial temperature is set to 100 °C. The Simple algorithm is selected in FLUENT.

#### 2.2.5. Prepare UDF and Apply Reaction Heat

The thermal decomposition temperature of ETPE is set at 453 K. According to reference [42], in stiu FT-IR was used to monitor the changes in the peak absorption of -NCO during the synthesis reaction of GAP-ETPE to calculate the kinetics of the reaction. On the basis of the Arrhenius Law Equation (10) and Eyring Law (11), the thermodynamic parameters of ETPE, such as the reaction activation energy ($E_{a}$), activation enthalpy (∆H) and activation entropy(ΔS), can be determined, and they are shown in Table 3. These parameters were programed into UDF and imported into FLUENT to prepare for the calculation of the ETPE ignition response time.

(10) $$\ln K=\ln A-\frac{E_{a}}{RT}$$

(11) $$ln\frac{K}{T}=-\frac{∆H}{RT}+\frac{∆S}{R}+\ln\frac{R}{Nh}$$

where *K* is the reaction rate constant at temperature *T*, min^−1^; $E_{a}$ is reaction activation energy, generally considered as a temperature-independent constant, J/mol or kJ/mol; *T* is the absolute temperature, *K*; ∆*H* is the activation enthalpy, also known as the heat of the reaction (when ∆*H* is negative, it is an exothermic reaction; when ∆*H* is positive, it is an endothermic reaction), kJ/mol; Δ*S* is activation entropy, J/(mol∙K); *R* is the molar gas constant, 8.314 J/(mol·K); *N* is the Avogadro’s constant (*N* = 6.02 × 10^23^); and *h* is the Planck’s constant (*h* = 6.62 × 10^−34^ J·s).

## 3. Results and Discussion

### 3.1. Analysis of Test Results

#### 3.1.1. Thermal Behavior of GAP-ETPE

**Figure 6:**
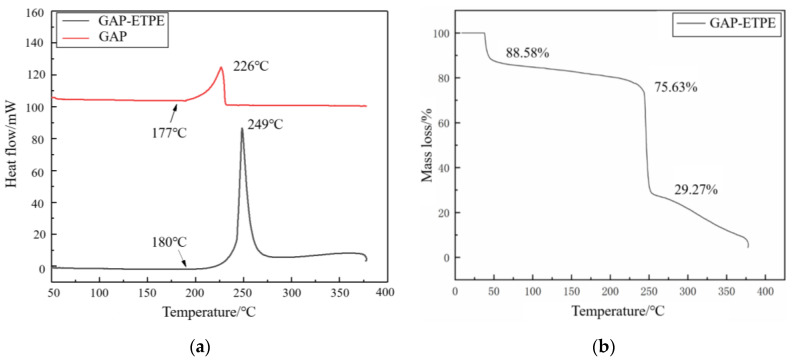
Thermal behavior of GAP-ETPE: (**a**) DSC curve of GAP and GAP-ETPE; (**b**) TG-DTG curve of GAP-ETPE.

The DSC and TG-DTG curves of pure GAP-ETPE at the heating rate of 5 °C/min are shown in Figure 6. As shown in Figure 6a, without any melting endothermic processes, the thermal decomposition process of GAP-ETPE shows a single exothermic peak at around 249 °C. With a molar reaction activation enthalpy of 1044 KJ/mol, the main decomposition process starts at around 180 °C and stops at around 280 °C. As shown in Figure 6b, the GAP-ETPE sample undergoes a main exothermic reaction during the second stage, which is confirmed by the DSC curves. The first decomposition stage starts at 40 °C and ends at 75 °C, with a mass loss of about 12%. The second decomposition stage starts at 220 °C and ends at 275 °C with a mass loss of approximately 46.4%. The mass loss in the second decomposition stage is closed to the total mass of the nitrogen content and can therefore be attributed to the release of N_2_ from the azide group via the cleavage of the azide bond. The third stage starts at about 290 °C and stops at 370 °C, which does not represent a significant exothermic effect. And the third stage experiences a nearly 30% mass loss due to the fracture of carbon backbones within the soft and hard segment molecular chains.

#### 3.1.2. Mechanical Sensitivity Analysis

**Figure 7:**
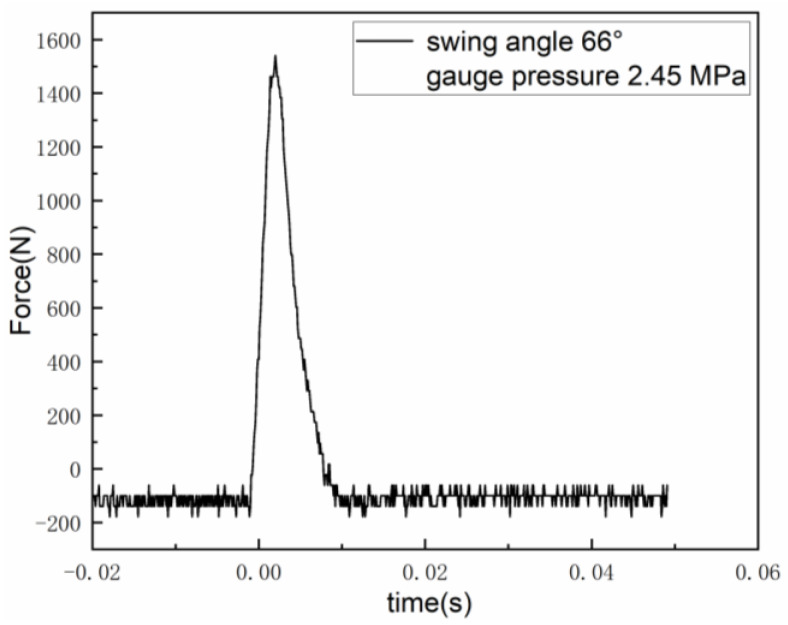
The impact force curve of pendulum of GAP-ETPE friction sensitivity.

The results of the impact and friction sensitivity tests for GAP-ETPE are shown in Table 4. From Table 4, it can be seen that the impact sensitivity value of GAP-ETPE is 0% and the friction sensitivity value is 15%. Based on the self-designed pendulum impact force testing system [43], the pendulum impact force was tested under the conditions of a 66° pendulum angle and 2.45 MPa overpressure, and the graph of the pendulum impact force versus time was obtained. From Figure 7, it can be seen that the peak value of the pendulum impact force reaches 1540 N. Since the diameter of the sliding column is 0.01 m, the action area is 7.85 × 10^−5^ m^2^. Finally, the ratio of the pendulum impact force to the effective area is 19.6 MPa, which is the shear stress of the GAP-ETPE sample, and the corresponding explosion rate of the friction sensitivity is 15%. 

### 3.2. Calculation Results and Safety Analysis

#### 3.2.1. Discussion and Safety Analysis of Temperature

**Figure 8:**
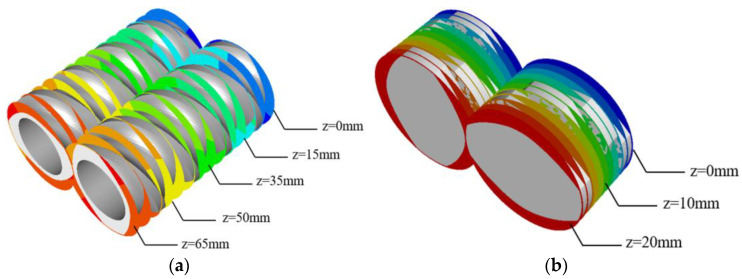
Monitoring plane sketch: (**a**) conveying element monitoring plane sketch; (**b**) kneading element monitoring plane sketch.

In order to understand the details of mixing and temperature variations in an intermeshing co-rotating twin screw, a number of monitoring planes have been selected. Figure 8 shows a sketch of an example plane for an intermeshing co-rotating twin screw. The coordinate origin is at the bottom of the screw and fifteen planes are selected as sample planes, i.e., plane 1 (Z = 0 mm)–plane 15 (Z = 65 mm) from the Z axis, to monitor the changes in temperature, pressure and shear stress at each plane.

**Figure 9:**
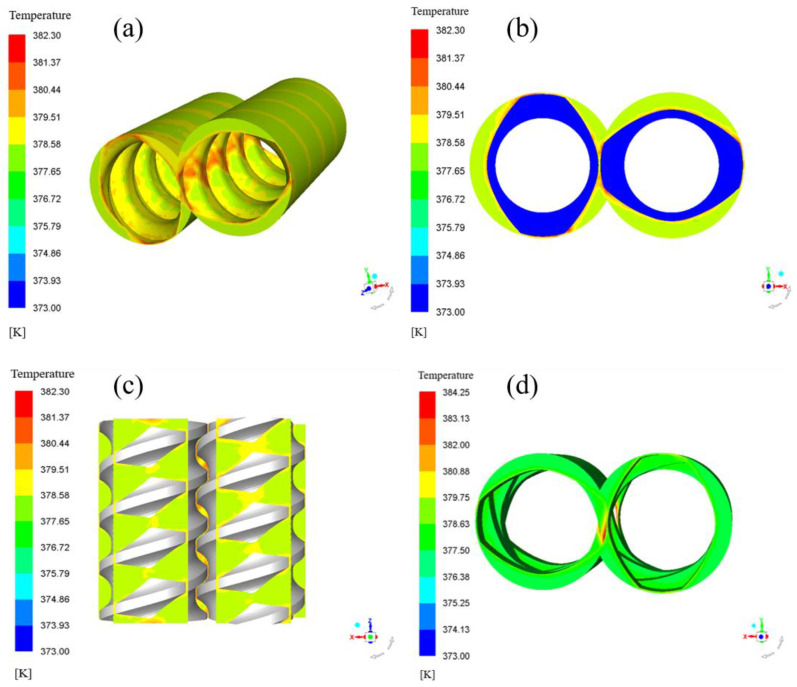
Temperature distribution at 40 rpm: (**a**) conveying element flow channel temperature distribution; (**b**) cross-section of temperature distribution of conveying element flow channel 16 mm from z axis; (**c**) profile of conveying element flow channel temperature distribution; (**d**) flow channel temperature distribution of kneading element.

**Figure 10:**
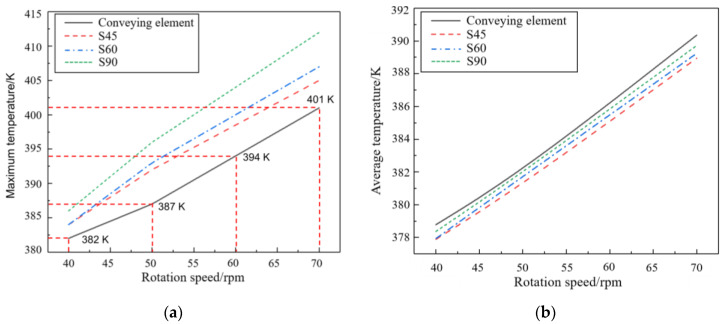
Effect of different rotation speeds on temperature: (**a**) maximum temperature variation curve in flow channel; (**b**) average temperature change curve in flow channel.

In order to reflect the fluid temperature changes in the twin-screw sleeve intuitively, the temperature distribution cloud diagram of the flow channel is processed by slicing, and the profile of ETPE temperature changes is obtained, as shown in Figure 9. It is found that the temperature rise points are mainly concentrated in the reactant mesh area, ETPE was obtained at the distance of 16 mm from Z axis, and it was found that the temperature rise point was concentrated on the contact surface between the screw and the flow channel and decreased gradually from inside to outside. With the increase in screw speed, the temperature in the flow channel is also changing under the conditions of viscous heat and friction. At 40 rpm, the temperature rises from 373 K to 382 K; at 50 rpm it rises to 387 K, at 60 rpm it rises to 394 K and at 70 rpm it rises to 401 K, as shown in Figure 10a. The twin-screw extruder is designed to be approximately 1710 mm and is evenly divided into eight barrels and one screw head, resulting in a length of approximately 190 mm for each segment. The simulated length of the conveying element is 65 mm in this paper, and it covers the three main reaction areas in the middle of the twin-screw extruder, mainly referring to the synthesis reaction area of conveying element barrels 4, 5 and 6. The research group conducted four temperature monitoring experiments at different speeds of 70, 60, 50 and 40 rpm, with two temperature monitoring points on each barrel. The monitoring temperatures of the conveying element for speeds of 70, 60, 50 and 40 rpm are approximately 132 °C (405 K), 124 °C (397 K), 119 °C (392 K) and 110 °C (383 K), respectively. The simulated predicted temperatures of the conveying element for the corresponding four rotational speeds are 128 °C (401 K), 121 °C (394 K), 114 °C (387 K) and 109 °C (382 K), respectively. From the comparison and analysis of predicted temperature results and actual monitoring temperature data, it can be seen that the temperature simulation results of the conveying element are in good agreement with the actual temperature monitoring results and have reliable accuracy.

In addition, as kneading elements S45, S60 and S90 have different intersection angles, the reactants in this mixing stage undergo strong shear, friction and compression effects, resulting in a significant conversion of mechanical energy into thermal energy. At this point, it is also easy to generate hot spots, as shown in the highest temperature in Figure 10a. At this stage, the highest temperature of the S90 kneading element, also known as the hot spot, is 4~12 K higher than the highest temperature of the conveying element, with a maximum temperature range of 386–413 K (113–140 °C). Compared with the initial thermal decomposition temperature of GAP-ETPE at 453 K (180 °C), the twin-screw extrusion process is still within the safe range at this time.

It can be seen that at low speed the temperature in the flow channel changes slowly. As the speed increases, the temperature clearly changes. The heat generated by mechanical friction between the conveying element and the flow channel is no longer the main source of energy change, and with the increase in shear stress, the viscous dissipation heat generated by GAP-ETPE gradually takes a dominant position. Figure 9 shows that the temperature rise of the conveying element and the kneading element is mainly concentrated in the meshing area, which is due to the shear and friction between the conveying element and the kneading element and the flow channel during the rotation process, resulting in the accumulation of energy and a higher temperature than other areas. Figure 10a shows that at the same rotational speed the maximum temperature of the kneading element is always slightly higher than that of the conveying element, and the order of the temperatures from highest to lowest is 90° kneading element, 60° kneading element, 45° kneading element and conveying element, but the average temperature in the flow channel is slightly higher than that of the kneading conveying element, as shown in Figure 10b. This is due to the fact that the shear, friction and extrusion are the main sources of heat in the engaging unit, with higher local hot spots, whereas the conveying unit is mainly reactive and exothermic. Therefore, the average temperature in the flow channel is slightly higher than that in the transport element.

#### 3.2.2. Discussion and Safety Analysis of Pressure

**Figure 11:**
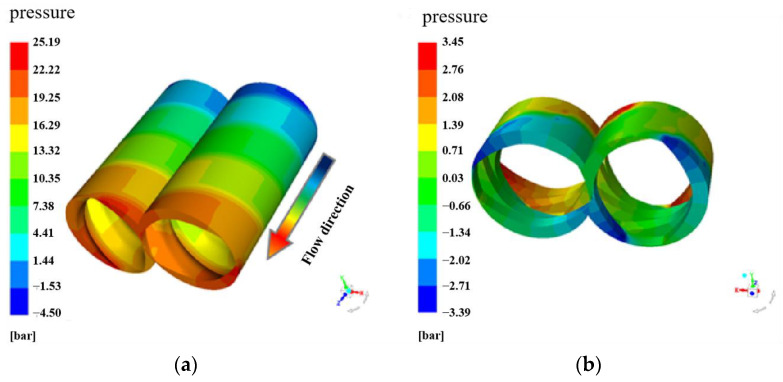
Pressure distribution at 40 rpm: (**a**) conveying element pressure distribution; (**b**) kneading element pressure distribution.

From the pressure cloud diagram of the conveying element in Figure 11, it can be seen that the pressure gradually increases along the extrusion direction and there is a higher pressure value at the outlet, the fluid is squeezed by the screw in the flow channel, and the pressure gradually increases. The inlet pressure is the lowest and the outlet pressure is the highest, reaching 25.19 bar, which is 8 times higher than that of the kneading element. When the pressure difference is large, the material flows faster and stays in the flow channel for a shorter time. Therefore, it can be concluded that the residence time of the material in the flow channel of the kneading element is longer than that in the flow channel of the conveying element, so that the material in the flow channel of the kneading element can be mixed more completely.

**Figure 12:**
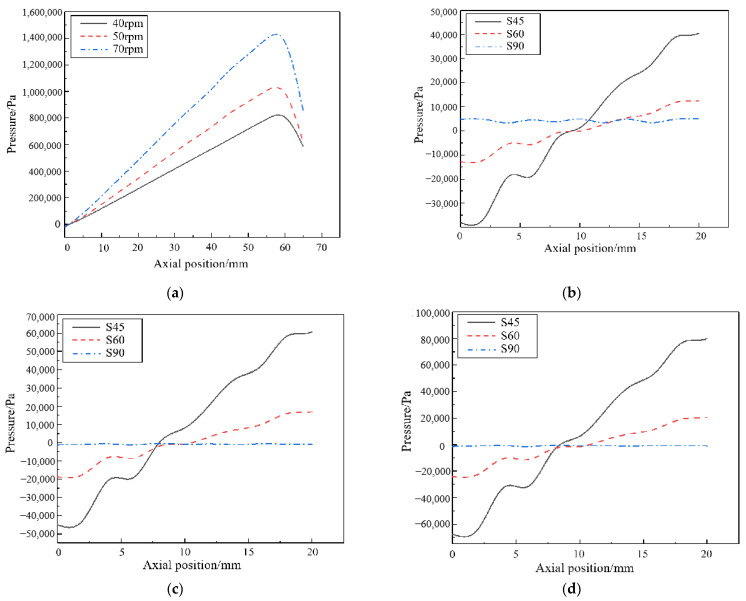
Effect of rotating speed on pressure: (**a**) effect of different rotating speeds on conveying element pressure; (**b**) effect of 40 rpm on kneading element pressure; (**c**) effect of 50 rpm on kneading element pressure; (**d**) effect of 70 rpm on kneading element pressure.

Figure 12a shows that the higher the speed of the conveying element, the higher the pressure in the flow channel and the greater the pressure difference between the inlet and outlet. The pressure at the inlet of the conveying element is negative, and the further away from the coordinate axis, the greater the pressure. Near the outlet, the pressure value drops sharply, and the higher the speed, the greater the drop, but it is still in accordance with the law that the higher the speed, the higher the pressure. Figure 12b–d shows that as the rotational speed of the kneading element increases, the maximum pressure in the flow channel is greater and the pressure difference between the inlet and outlet is greater. However, the pressure of the different types of kneading elements is also different, with S45 being the largest, S60 the second-largest and S90 the smallest. In S90, the material flow speed is slow, the residence time is long and the mixing is more sufficient. It can be seen from Figure 12d that the maximum fluid pressure of GAP-ETPE is 0.08 MPa during the whole process of twin-screw transport and engagement, which is much lower than the overpressure of 2.45 MPa of the friction sensitive device. This safety factor of GAP-ETPE in the process of twin-screw extrusion is relatively high.

#### 3.2.3. Discussion and Safety Analysis of Shear Stress and Velocity

**Figure 13:**
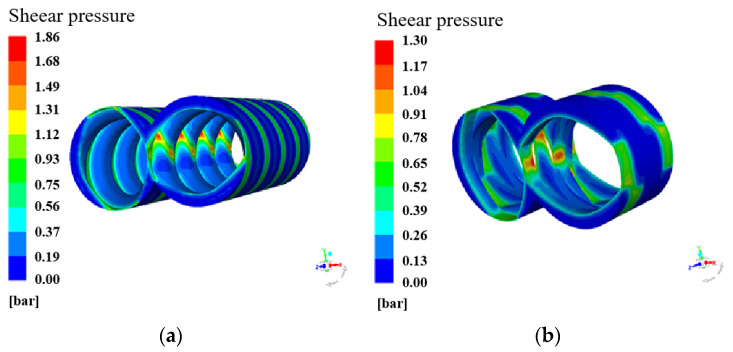
Shear stress distribution at 40 rpm: (**a**) conveying element shear stress distribution; (**b**) kneading element shear stress distribution.

**Figure 14:**
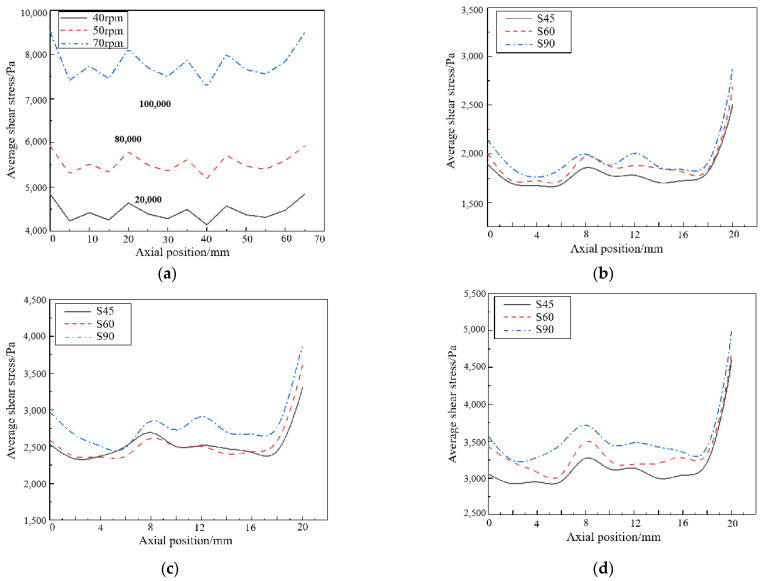
Effect of rotating speed on shear stress: (**a**) effect of different rotating speeds on conveying element shear stress; (**b**) effect of 40 rpm on shear stress of kneading element; (**c**) effect of 50 rpm on shear stress of kneading element; (**d**) effect of 70 rpm on shear stress of kneading element.

From the shear stress cloud diagram in Figure 13 of the conveying element and the kneading element, the shear stress is greatest in the meshing area, there is a higher shear stress near the top of the screw, and there is a lower shear stress area near the screw groove. From the top of the screw to the root of the screw groove, the material is subjected to shear stresses from high to low. Figure 14 shows that the shear stress of the conveying element is higher than that of the kneading element when the rotational speed is fixed, the shear stress becomes larger and larger as the rotational speed increases, and the dispersing and mixing ability is stronger. Among the kneading elements, the 90° kneading element has the strongest dispersing and mixing ability, the 60° kneading element takes the second place and the 45° kneading element is the worst. As the rotational speed increases, the shear stress in the flow channel of the impeller increases and has a greater variation at the inlet and outlet. It can be seen from Figure 14d that the maximum shear stress of GAP-ETPE is about 0.005 MPa during the whole process of twin-screw transport and engagement, which is much smaller than the impact force of 19.6 MPa of the friction pendulum under the gauge pressure of 2.5 MPa. Therefore, GAP-ETPE has a high degree of safety in the twin-screw extrusion process.

**Figure 15:**
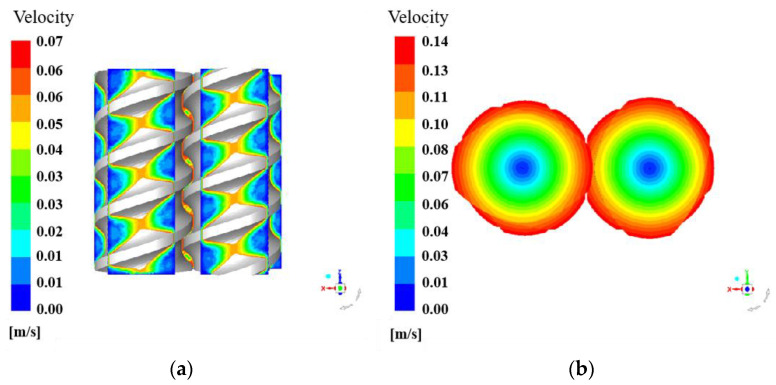
Velocity distribution at 40 rpm: (**a**) conveying element velocity distribution profile; (**b**) kneading element velocity distribution.

Figure 15a shows that the speed of the conveying element is greatest in the meshing area, and then the speed of the fluid contact area with the screw edge is also greater, which is conducive to the complete mixing of the materials, and the fluid area of the kneading element also follows this rule. Figure 15b shows the velocity distribution of the mixing element itself, which increases from the center to either side. The greater the distance, the greater the velocity.

#### 3.2.4. Discussion and Safety Analysis of Response Time

**Figure 16:**
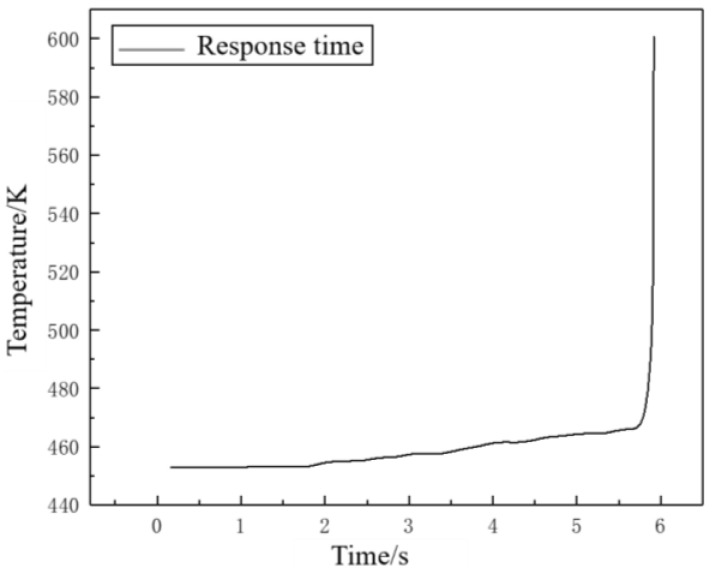
Ignition response time chart.

The thermal decomposition temperature of GAP-ETPE is 453 K (180 °C). The thermophysical parameters of GAP-ETPE in Table 3 are compiled and imported into UDF. When the initial temperature is 453 K, the temperature is unchanged within 1 s, and the temperature increases continuously from 1 s to 5.9 s without any obvious change, but the temperature increases sharply at 5.91 s in Figure 16. It can be seen that ETPE is subject to thermal decomposition. Therefore, to ensure the normal operation of the GAP-ETPE twin-screw extruder, the temperature in the TSE should be lower than 453 K while considering the material mixing uniformity; when the temperature in the TSE reaches 453 K, the mixing equipment should be cooled within 5.9 s to ensure the safety of the equipment. This provides theoretical guidance for the safety design of the GAP-ETPE twin-screw extruder.

## 4. Conclusions

In this paper, a three-dimensional numerical simulation method is used to study the material flow and temperature change of GAP-ETPE during the reactive extrusion of conveying and kneading elements. The following conclusions are drawn: 

(1) The heating of the polymer GAP-ETPE comes from the heat conduction in the flow channel, the shear friction between the screw and the flow channel and the heat dissipation caused by the viscosity consumption of the polymer GAP-ETPE. At low speeds, the heat conduction and shear friction in the flow channel are the main factors. As the speed increases, the viscosity consumption and heat dissipation caused by GAP-ETPE gradually become dominant. 

(2) At the same speed, the maximum temperature of the kneading element is always slightly higher than that of the conveying element. The order of temperature is 90° kneading element > 60° kneading element > 45° kneading unit > conveying unit, but the average temperature in the flow channel is always slightly higher than the average temperature of the kneading unit. The shear stress of the screw is higher than that of the kneader. As the speed increases, the shear stress increases, resulting in a greater dispersing and mixing ability.

(3) The higher the speed of the conveying and kneading elements, the greater the maximum pressure in the flow channel and the greater the pressure difference between the inlet and outlet. The pressure of the conveying element is higher than that of the kneading element, but the pressure of the kneading element varies with different staggered angles. S45 has the largest pressure change, S60 has the second-largest and S90 has the smallest pressure change. Among them, the pressure difference between the inlet and outlet of the S90 kneading element is the smallest, the residence time is longer and the mixing is more thorough.

(4) The thermal decomposition temperature of GAP-ETPE is 453 K (180 °C). When the initial temperature is 453 K, the temperature is basically unchanged within 1 s, and the temperature rises continuously from 1 s to 5.9 s without any obvious change, but the temperature rises sharply at 5.91 s. In addition, the internal temperature rises of GAP-ETPE synthesized by a twin-screw extruder with a diameter of 26 mm are relatively small under the process conditions of 40–70 rpm, which is much lower than the thermal decomposition temperature of GAPE-ETPE at 180 °C. Therefore, the production process for synthesizing GAP-ETPE through twin-screw reactive extrusion is generally safe.

## Figures and Tables

**Table 1:** Twin-screw structure parameters.

Screw Diameter/mm	Screw Clearance/mm	Lead Range/mm	Sleeve Length/mm	Screw Length/mm
26	0.4	32	65	65

**Table 2:** Structure parameters of kneading element.

Name	Disc Thickness (Number/mm)	Stagger Angle/°	Involute End Circle Diameter/mm	Minimum Tooth Width/mm	Maximum Actual Tooth Width/mm
S90	5/4	90	13	1.49	1.63
S60	5/4	60	13	1.49	1.63
S45	5/4	45	13	1.49	1.63

**Table 3:** Thermodynamic parameters of ETPE.

$E_{a}$(kJ/mol)	*A*	∆H(kJ/mol)	∆S(J/mol^−1^·K^−1^)
108.996	1.7 × 10^10^	1044	−59.397

**Table 4:** Test results of impact sensitivity and friction sensitivity of GAP-ETPE propellant.

Sample	Impact Sensitivity/%	Friction Sensitivity/%
GAP-ETPE	0	15

## Data Availability

The data presented in this study are openly available.

## Nomenclature

𝑝	Pressure
$\tau_{ij}$	Stress tensor component
t	Time
ρ	Density
𝑘	Thermal conductivity
$C_{p}$	Specific heat capacity
𝜂	Viscosity
$\dot{\gamma }$	Shear rate
K	Reaction rate constant at temperature
$E_{a}$	Reaction activation energy
∆H	Decomposition reaction exothermic
*R*	Molar gas constant
∆S	Activation entropy
μ	Dynamic viscosity
λ	Second viscosity
$f_{x}$	Unit mass forces in X direction
$f_{y}$	Unit mass forces in Y direction
$f_{z}$	Unit mass forces in Z direction
$u_{x}$	Velocity component in X direction
$u_{y}$	Velocity component in Y direction
$u_{z}$	Velocity component in Z direction
